# Identification of a Novel Cobamide Remodeling Enzyme in the Beneficial Human Gut Bacterium Akkermansia muciniphila

**DOI:** 10.1128/mBio.02507-20

**Published:** 2020-12-08

**Authors:** Kenny C. Mok, Olga M. Sokolovskaya, Alexa M. Nicolas, Zachary F. Hallberg, Adam Deutschbauer, Hans K. Carlson, Michiko E. Taga

**Affiliations:** a Department of Plant & Microbial Biology, University of California, Berkeley, Berkeley, California, USA; b Environmental Genomics and Systems Biology Division, Lawrence Berkeley National Laboratory, Berkeley, California, USA; Baylor College of Medicine

**Keywords:** *Akkermansia muciniphila*, cobalamin, cobamide remodeling, corrinoids, vitamin B12

## Abstract

The beneficial human gut bacterium Akkermansia muciniphila provides metabolites to other members of the gut microbiota by breaking down host mucin, but most of its other metabolic functions have not been investigated. *A. muciniphila* strain Muc^T^ is known to use cobamides, the vitamin B_12_ family of cofactors with structural diversity in the lower ligand. However, *A. muciniphila* Muc^T^ is unable to synthesize cobamides *de novo*, and the specific forms that can be used by *A. muciniphila* have not been examined. We found that the levels of growth of *A. muciniphila* Muc^T^ were nearly identical with each of seven cobamides tested, in contrast to nearly all bacteria that had been studied previously. Unexpectedly, this promiscuity is due to cobamide remodeling—the removal and replacement of the lower ligand—despite the absence of the canonical remodeling enzyme CbiZ in *A. muciniphila*. We identified a novel enzyme, CbiR, that is capable of initiating the remodeling process by hydrolyzing the phosphoribosyl bond in the nucleotide loop of cobamides. CbiR does not share similarity with other cobamide remodeling enzymes or B_12_-binding domains and is instead a member of the apurinic/apyrimidinic (AP) endonuclease 2 enzyme superfamily. We speculate that CbiR enables bacteria to repurpose cobamides that they cannot otherwise use in order to grow under cobamide-requiring conditions; this function was confirmed by heterologous expression of *cbiR* in Escherichia coli. Homologs of CbiR are found in over 200 microbial taxa across 22 phyla, suggesting that many bacteria may use CbiR to gain access to the diverse cobamides present in their environment.

## Abstract (precis)

Cobamides, comprising the vitamin B_12_ family of cobalt-containing cofactors, are required for metabolism in all domains of life, including most bacteria. Cobamides have structural variability in the lower ligand, and selectivity for particular cobamides has been observed in most organisms studied to date.

## INTRODUCTION

The human gut microbiota is composed of diverse communities of microbes that play important roles in human health (1–4). Disruption of the composition of the microbiota, known as dysbiosis, is associated with numerous disease states (5–9). While the immense complexity and interindividual variability of the microbiota have made it challenging to identify the specific functions of most community members, particular taxa are starting to be linked to health and disease (10–12), with the bacterium Akkermansia muciniphila recently emerging as a beneficial microbe due to its distinctive metabolic capabilities (13, 14).

**FIG 1:**
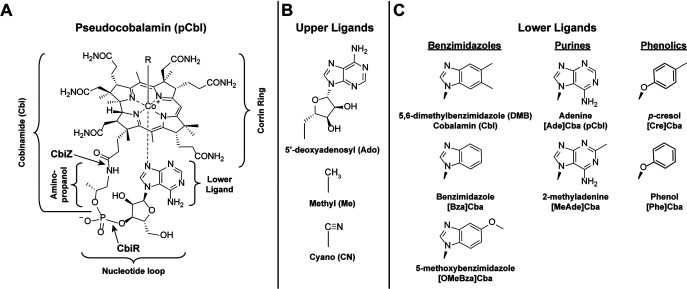
Cobamide structures. (A) Structure of pCbl. All cobamides are composed of a corrin ring containing a central cobalt ion and an upper (R) and lower ligand. In pCbl, the lower ligand is adenine. The lower ligand and the ribose and phosphate moieties comprise the nucleotide loop, which is covalently attached to the corrin ring via an aminopropanol linker. The bonds hydrolyzed by the CbiZ amidohydrolase and the CbiR phosphodiesterase are indicated with arrows. The part of the molecule comprising Cbi is shown. Cobamides and their corrin-containing biosynthetic precursors and degradation products are together known as corrinoids. (B) Upper ligands (R) in cobamides, the catalytic center of the cofactor; prefixes used in the text to denote the upper ligand are shown in parentheses. (C) The three chemical classes of lower ligands present in cobamides. The structures of the seven cobamide lower ligands used in this study are shown. The names of the lower ligand base and abbreviations used for the corresponding cobamides are given below the structures.

*A. muciniphila* is thought to benefit the host by inducing mucus production, improving gut barrier function, and stimulating a health-promoting inflammatory response (15–24). *A. muciniphila* is one of few bacterial species capable of using mucin, the main component of mucus, as a sole carbon, nitrogen, and energy source (25). Mucin degradation products released by *A. muciniphila* are used as carbon sources by butyrate-producing bacteria and likely other bacteria, and for this reason *A. muciniphila* is thought to be a keystone species in the gut (26, 27). In addition to providing metabolites to neighboring microbes, in coculture *A. muciniphila* can use a cobamide cofactor, pseudocobalamin (pCbl; Fig. 1A), provided by Eubacterium hallii for the production of propionate (26). Both butyrate and propionate positively affect host metabolism and immune function (28–30). Along with the enzyme methylmalonyl-coenzyme A mutase (MCM), which is required for propionate production, nearly all *A. muciniphila* strains have homologs of three other cobamide-dependent enzymes, methionine synthase (MetH), ribonucleotide reductase (NrdJ), and epoxyqueuosine reductase (QueG) (31).

Cobamides are a family of cobalt-containing corrinoid cofactors that include B_12_ (cobalamin [Cbl]), an essential micronutrient for humans. Cobamides are required by organisms in all domains of life but are synthesized by only a subset of prokaryotes (32–34). While some strains of *A. muciniphila* have been shown or are predicted to produce cobamides *de novo*, the type strain, Muc^T^, is incapable of *de novo* cobamide production (31). Instead, strain Muc^T^ and most other *A. muciniphila* strains are predicted to be capable of cobinamide (Cbi; Fig. 1A) salvaging (31, 34), a process in which a cobamide is synthesized from the late precursor Cbi (35). Thus, the four cobamide-dependent metabolic pathways present in *A. muciniphila* function in most strains, including Muc^T^, only when a cobamide or a late precursor such as Cbi is provided by another organism. Several other human gut bacterial species have similarly been found to use cobamide cofactors but to be unable to produce them *de novo*, including Bacteroides fragilis, Bacteroides thetaiotaomicron, Bacteroides vulgatus, Clostridioides difficile, Enterococcus faecalis, Escherichia coli, and Parabacteroides distasonis (36–40). In addition to these specific examples, genomic analysis suggests that dependence on cobamide-producing microbes is widespread in the gut and other environments: 58% of human gut bacteria and 49% of all sequenced bacteria are predicted to use cobamides but to lack the capacity to produce them *de novo* (34).

A feature that sets cobamides apart from other enzyme cofactors is that different microbes produce structurally distinct cobamides (41). This variability is mostly limited to the lower ligand, which can be benzimidazolyl, purinyl, or phenolyl bases (Fig. 1C). Individual cobamide-producing bacteria typically synthesize only one type of cobamide, but microbial communities have been found to contain four to eight different cobamides or cobamide precursors (42–45). A study of 20 human subjects showed that the human gut is dominated by the purinyl class of cobamides, with benzimidazolyl and phenolyl cobamides and Cbi also present (42). The structural diversity in cobamides impacts growth and metabolism, as most organisms studied to date, including both cobamide producers and auxotrophs, are selective in their cobamide use (39, 46–54). For example, the human gut bacterium B. thetaiotaomicron can use benzimidazolyl and purinyl cobamides but not phenolyl cobamides (37); Dehalococcoides mccartyi is selective for particular benzimidazolyl cobamides (55, 56); many eukaryotic algae prefer Cbl to pCbl (57); and Sporomusa ovata requires phenolyl cobamides (58). Thus, microbes that depend on cobamides produced by others may struggle to grow in environments lacking their preferred cobamides. However, some organisms have evolved mechanisms for acquiring the specific cobamides that function in their metabolism. For example, bacterial cobamide uptake can be somewhat selective, as shown in a study in B. thetaiotaomicron (37). Another strategy used by some microbes is cobamide remodeling, i.e., the removal and replacement of the lower ligand.

Cobamide remodeling was first described in Rhodobacter sphaeroides (59) but has also been observed in the bacteria *D. mccartyi* and Vibrio cholerae and the algae Pavlova lutheri and Chlamydomonas reinhardtii (45, 48, 56, 57). In each case, cobamide remodeling enables the organism to repurpose a cobamide that supports growth poorly. In R. sphaeroides, the cobamide remodeling process is initiated by the enzyme CbiZ, which hydrolyzes the amide bond adjacent to the aminopropanol linker (Fig. 1A) (59); in subsequent steps, cobamide biosynthesis is completed with a different lower ligand via the activity of six gene products, most of which are also required for Cbi salvaging. *In vitro*, R. sphaeroides CbiZ hydrolyzes pCbl but not Cbl (59). This specificity is thought to drive the conversion of pCbl, a cofactor that R. sphaeroides cannot use, into Cbl, which functions in its metabolism. *D. mccartyi* also has homologs of *cbiZ* (56), while cobamide remodeling in V. cholerae was recently shown to involve the cobamide biosynthesis enzyme CobS (48). The genes required for cobamide remodeling in algae have not been identified. Nevertheless, cobamide remodeling has not been found to be widespread; it has rarely been observed in cultured bacteria, and over 90% of corrinoid-requiring bacteria, including *A. muciniphila*, lack a homolog of *cbiZ* (34).

Here, we show that *A. muciniphila* strain Muc^T^ is able to grow equivalently when provided a variety of cobamides. We found that this lack of selectivity is due to the unexpected ability of *A. muciniphila* to remodel cobamides. We identified a previously uncharacterized phosphodiesterase in *A. muciniphila* that we named CbiR and which initiates the remodeling process by hydrolyzing cobamides. Heterologous expression in E. coli shows that CbiR expands access to a cobamide that does not otherwise support growth. Homologs of CbiR are present in the genomes of microbes in diverse habitats from 22 phyla, and phylogenetic analysis establishes CbiR as a new, distinct clade within the AP endonuclease 2 superfamily. These observations enhance the understanding of the metabolic roles of *A. muciniphila* and improve our ability to predict cobamide-dependent physiology in other bacteria.

## RESULTS

### *A. muciniphila* strain Muc^T^ salvages Cbi to produce pCbl.

**FIG 2:**
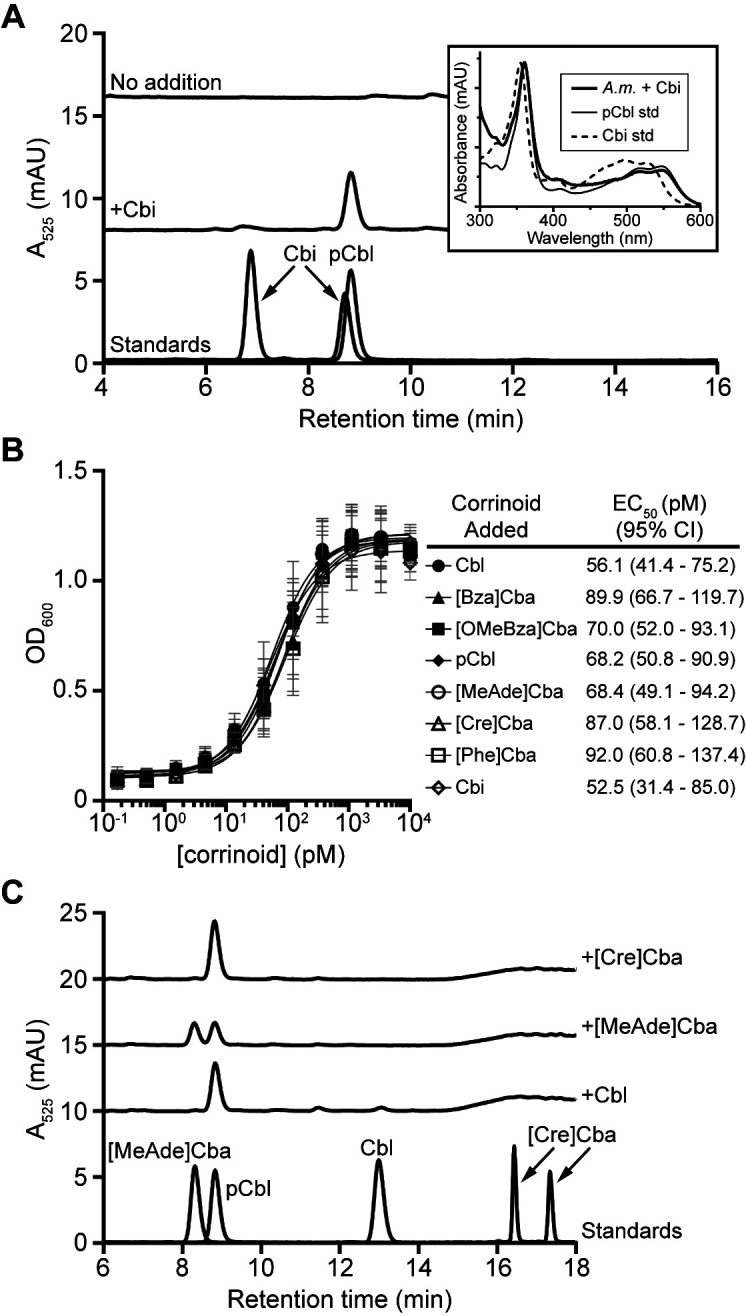
*A. muciniphila* strain Muc^T^ can salvage Cbi and remodel cobamides. (A) HPLC analysis of corrinoid extractions from *A. muciniphila* grown with methionine in the presence or absence of 10 nM Cbi for 72 h showed that *A. muciniphila* can salvage Cbi. Standards for Cbi and pCbl are shown at the bottom. Note that Cbi appears as two peaks. Comparison of UV-Vis spectra (inset) of the HPLC peaks at 8.8 min shows that the corrinoid produced by *A. muciniphila* (*A.m.*) grown with Cbi (thick line) is similar to a pCbl standard (std) (thin line) and not a Cbi standard (dashed line). Spectra were normalized to each other based on their maxima to aid comparison. mAU, milliarbitrary units. (B) *A. muciniphila* growth under methionine-deplete conditions. The OD_600_ values shown were determined for saturated cultures after 29 h of growth with the indicated concentrations of each corrinoid. EC_50_ values and the corresponding 95% confidence intervals for each corrinoid are given in the table. Data points and error bars represent means and standard deviations, respectively, of results from three biological replicates. The results are representative of three independent experiments. (C) HPLC analysis of corrinoid extractions from *A. muciniphila* grown with 10 nM Cbl, [MeAde]Cba, or [Cre]Cba for 72 h showed that *A. muciniphila* remodels cobamides to pCbl. Cobamide standards are shown at the bottom, with [Cre]Cba appearing as two peaks.

*A. muciniphila* strain Muc^T^ lacks most of the genes required for cobamide synthesis and does not produce cobamides *de novo* (31), but it is predicted to be capable of Cbi salvaging (34). To test this prediction, we extracted corrinoids from *A. muciniphila* cultured with and without Cbi and analyzed the corrinoid composition of the samples by high-performance liquid chromatography (HPLC). Cultured without Cbi, no corrinoids were detected in the extractions (Fig. 2A). However, when Cbi was added to the growth medium, a cobamide with the same retention time and a UV-visible light (UV-Vis) spectrum nearly identical to that of pCbl was detected by HPLC (Fig. 2A). Mass spectrometry (MS) analysis confirmed that this cobamide is pCbl (see Fig. S1 in the supplemental material).

10.1128/mBio.02507-20.1 FIG S1 The corrinoid produced by *A. muciniphila* cultured with Cbi was purified by HPLC and analyzed by MS. The structure and *m*/*z* values predicted for CN-pCbl are shown for comparison. Download FIG S1, PDF file, 0.2 MB. Copyright © 2020 Mok et al. 2020 Mok et al. This content is distributed under the terms of the Creative Commons Attribution 4.0 International license.

### *A. muciniphila* strain Muc^T^ does not show cobamide selectivity.

Having established that *A. muciniphila* strain Muc^T^ cannot synthesize cobamides without the addition of a precursor, we next examined which cobamides it is capable of using by measuring growth in the presence of various cobamides under a cobamide-requiring condition. *A. muciniphila* strain Muc^T^ has four cobamide-dependent enzymes; however, MCM is not required for laboratory growth (60), QueG function does not affect growth in other bacteria (61), and the cobamide-independent ribonucleotide reductase NrdDG likely renders NrdJ nonessential. Because *A. muciniphila* lacks a homolog of the cobamide-independent methionine synthase MetE (34), growth in the absence of methionine is expected to be dependent on the cobamide-dependent methionine synthase MetH. Thus, growth in methionine-deplete medium is predicted to require cobamide addition. We found this to be the case, as addition of Cbi or any of the seven cobamides tested was necessary to support growth of *A. muciniphila* (Fig. 2B). Surprisingly, however, *A. muciniphila* showed essentially no cobamide selectivity, with less than 2-fold variations in the cobamide concentrations resulting in half-maximal growth (EC_50_) (Fig. 2B).

### *A. muciniphila* strain Muc^T^ remodels cobamides to pCbl.

The ability of all of the tested cobamides to support nearly identical levels of growth of *A. muciniphila* could have been due to promiscuity in its cobamide-dependent methionine synthase. Alternatively, *A. muciniphila* might remodel cobamides, despite the absence of a homolog of *cbiZ* in its genome. If cobamide remodeling occurs in *A. muciniphila*, exogenously supplied cobamides would be altered by the bacterium. Therefore, we extracted corrinoids from *A. muciniphila* cultures supplemented with Cbl, [Cre]Cba, or [MeAde]Cba to determine whether the added cobamides could be recovered. HPLC analysis revealed that none of the Cbl or [Cre]Cba and only half of the [MeAde]Cba remained in the extractions. This loss of the added cobamide coincided with the appearance of a new cobamide that coeluted with pCbl (Fig. 2C). MS analysis confirmed that this cobamide was indeed pCbl (Fig. S2). These results demonstrate that *A. muciniphila* remodels cobamides to pCbl.

10.1128/mBio.02507-20.2 FIG S2 The corrinoid produced by *A. muciniphila* cultured with Cbl was purified by HPLC and analyzed by MS. The structure and *m*/*z* values predicted for CN-pCbl are shown for comparison. Download FIG S2, PDF file, 0.2 MB. Copyright © 2020 Mok et al. 2020 Mok et al. This content is distributed under the terms of the Creative Commons Attribution 4.0 International license.

### Identification and characterization of a novel cobamide remodeling enzyme in *A. muciniphila*.

**FIG 3:**
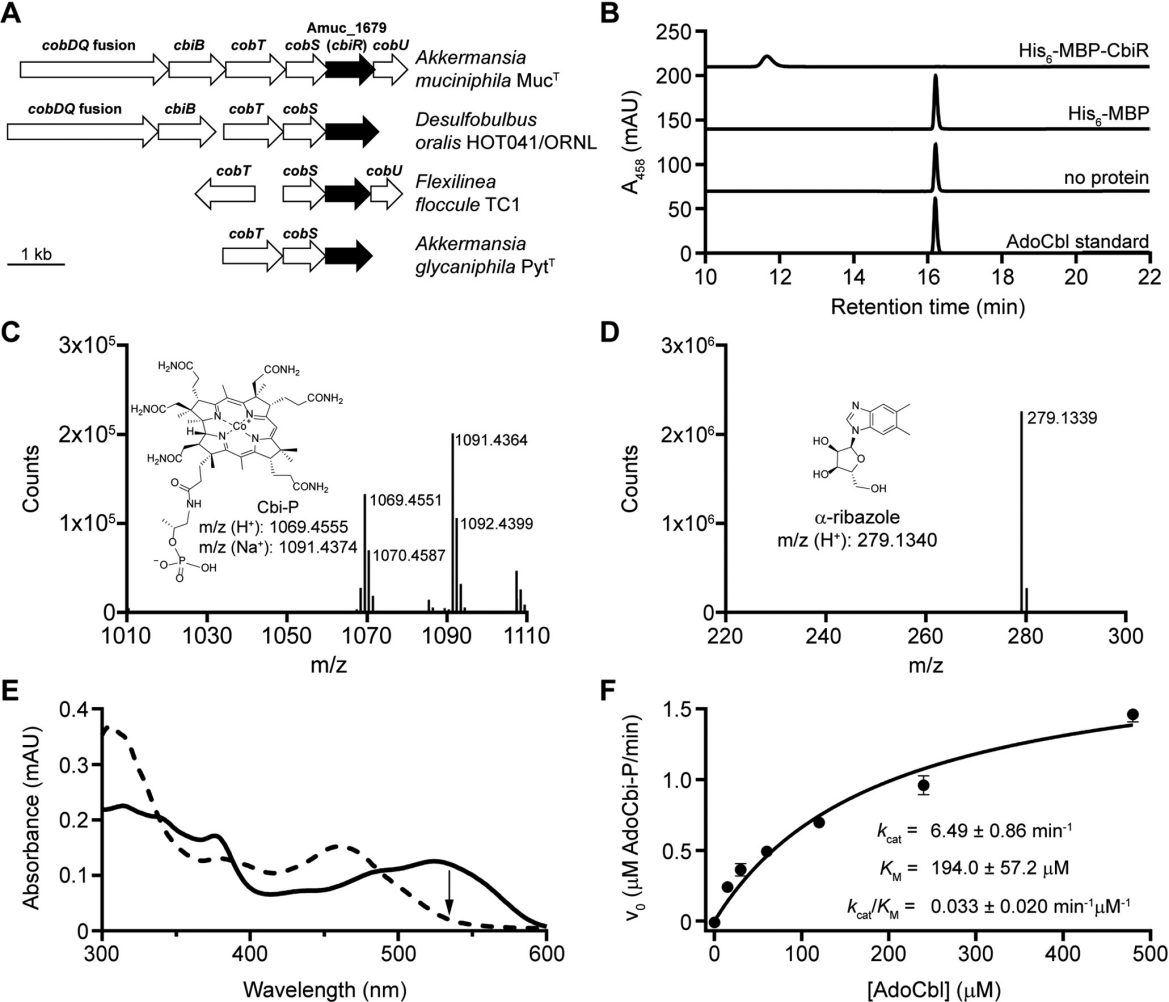
Purified CbiR hydrolyzes AdoCbl to form AdoCbi-P and α-ribazole *in vitro*. (A) *A. muciniphila* Amuc_1679 (*cbiR*) and homologs in other bacteria (black arrows) are located near cobamide biosynthesis genes (white arrows). An expanded genomic comparison is shown in Fig. S3. (B) Purified CbiR converts AdoCbl to another corrinoid compound *in vitro* in the absence of oxygen. Results of HPLC analysis of reaction mixtures containing 10 μM AdoCbl incubated for 4 h with 0.1 μM His_6_-MBP-CbiR, 0.1 μM His_6_-MBP, or no protein are shown. An AdoCbl standard is shown at bottom. (C) The corrinoid product of His_6_-MBP-CbiR was purified by HPLC, exposed to light to remove the adenosyl upper ligand, and analyzed by MS. The structure and *m*/*z* values predicted for Cbi-P are shown for comparison. (D) The second product of the *in vitro* reaction with His_6_-MBP-CbiR and AdoCbl was purified by HPLC and analyzed by MS. The structure and *m*/*z* values predicted for α-ribazole are shown for comparison. (E) Comparison of the UV-Vis spectra before completion (solid line) and after completion (dashed line) of the reaction of His_6_-MBP-CbiR with 30 μM AdoCbl shows a decrease in absorbance at 534 nm (arrow). (F) Michaelis-Menten kinetic analysis of His_6_-MBP-CbiR with AdoCbl. Reactions contained 0.3 μM His_6_-MBP-CbiR. Points and error bars represent means and standard deviations, respectively. Kinetic constants were determined from two independent experiments, each performed with three technical replicates.

The identification of cobamide remodeling activity despite the absence of a *cbiZ* homolog in the genome suggested that a novel enzyme capable of hydrolyzing cobamides is present in *A. muciniphila*. We reasoned that the gene encoding this enzyme might be located near the cobamide biosynthesis and salvaging genes *cobDQ*, *cbiB*, *cobT*, *cobS*, and *cobU*, some or all of which would be required for completion of the remodeling process. These five genes are found at a single locus in the *A. muciniphila* genome that also contains an open reading frame (ORF) with unknown function, annotated as Amuc_1679 (GenBank accession no. ACD05497.1) (Fig. 3A). Amuc_1679 is predicted to encode a protein with a conserved (β/α)_8_ TIM barrel domain from the apurinic/apyrimidinic (AP) endonuclease 2 superfamily (pfam01261). This superfamily is composed of several enzymes, including endonuclease IV, which hydrolyzes phosphodiester bonds at AP sites in DNA (62). The proximity of Amuc_1679 to genes involved in cobamide biosynthesis and the presence of a phosphodiester bond connecting the lower ligand to the aminopropanol linker suggested that Amuc_1679 might play a role in cobamide biology in *A. muciniphila*. Further, homologs of this gene in other bacteria are also found in loci containing similar cobamide biosynthesis enzymes (Fig. 3A; see also Fig. S3).

10.1128/mBio.02507-20.3 FIG S3 Expanded list of homologs of Amuc_1679 (*cbiR*, red arrows). Species and strain names are given, with phylum names in parentheses. “RS” denotes a predicted cobalamin riboswitch. The lengths and positions of the ORFs are drawn to scale. Download FIG S3, PDF file, 0.4 MB. Copyright © 2020 Mok et al. 2020 Mok et al. This content is distributed under the terms of the Creative Commons Attribution 4.0 International license.

To determine whether Amuc_1679 encodes an enzyme that can hydrolyze cobamides, we overexpressed and purified Amuc_1679 for analysis of its activity *in vitro*. N-terminal hexahistidine (His_6_) and maltose-binding protein (MBP) tags were added to aid in the purification and to increase the solubility of the protein, respectively (Fig. S4A). First, we tested whether a new product was formed when the protein was incubated with coenzyme B_12_ (AdoCbl), an active cofactor form of Cbl. We observed complete conversion of AdoCbl to a new corrinoid compound in reactions performed under anaerobic conditions (Fig. 3B). MS analysis showed that two reaction products, cobinamide-phosphate (Cbi-P) and α-ribazole, were formed, indicating hydrolysis of the phosphoribosyl bond of AdoCbl (Fig. 3C and D). Notably, Amuc_1679 targets a bond distinct from the enzyme CbiZ (Fig. 1A) (59). Synthesis of the cobamide from Cbi-P might be a more efficient remodeling process as it would bypass the need to rebuild the aminopropanol linker. In keeping with the tradition of naming cobamide biosynthesis and remodeling enzymes with a “Cbi” prefix, we refer to Amuc_1679 as CbiR here.

10.1128/mBio.02507-20.4 FIG S4 Biochemical characterization of His_6_-MBP-CbiR. (A) SDS-PAGE gel of purification of His_6_-MBP-CbiR with Ni-NTA resin. Lane 1, cell lysate; lane 2, flowthrough; lanes 3 to 5, wash fractions; lanes 6 to 9, elution fractions. His_6_-MBP-CbiR has a predicted molecular weight of 77 kDa. (B) *In vitro* characterization of His_6_-MBP-CbiR. The reaction rates were determined for 0.3 μM His_6_-MBP-CbiR incubated with 30 μM AdoCbl. The standard reaction mixture used as described throughout the manuscript (labeled “-O_2_”) contained 50 mM Tris (pH 8.45 to 8.55) and 1 mM DTT, and was performed in an anaerobic chamber. Activity was also measured in ambient O_2_ (+O_2_), with DTT omitted (-DTT), with 3 μM EDTA added (+EDTA), and in the absence of His_6_-MBP-CbiR (no protein). The reaction was monitored by measuring *A*_534_ levels over time. Lines and error bars represent means and standard deviations, respectively. (C) The corrinoid product of incubation of His_6_-MBP-CbiR with MeCbl was purified by HPLC, exposed to light to remove the methyl upper ligand, and analyzed by MS. The structure and *m*/*z* values predicted for Cbi-P are shown for comparison. (D) Michaelis-Menten kinetic analysis of His_6_-MBP-CbiR with MeCbl. His_6_-MBP-CbiR was tested at 0.3 μM, and the reaction was monitored by measuring the decrease in absorbance at 527 nm (*A*_527_). The experiment was performed twice with similar results. Data from a representative experiment performed with three technical replicates are shown. Kinetic constants were calculated using data from all six replicates. Download FIG S4, PDF file, 0.6 MB. Copyright © 2020 Mok et al. 2020 Mok et al. This content is distributed under the terms of the Creative Commons Attribution 4.0 International license.

We were able to monitor CbiR activity continuously by measuring the rate of decrease in absorbance at 534 nm (*A*_534_), as the reaction is characterized by a change in the UV-Vis spectrum that reflects the loss of AdoCbl and formation of AdoCbi-P (Fig. 3E). With this method, we found that the reaction proceeded only in the absence of oxygen and, additionally, that the reaction required the reducing agent dithiothreitol (DTT) and was inhibited by the metal chelator EDTA (Fig. S4B). Although other members of the AP endonuclease 2 superfamily require metals (63–80), CbiR is the only member found to be sensitive to oxygen.

Using the same method, we determined the reaction kinetics of His_6_-MBP-CbiR under steady-state conditions over a range of AdoCbl concentrations (Fig. 3F). On the basis of a fit to the Michaelis-Menten model, the reaction of His_6_-MBP-CbiR with AdoCbl exhibited *K_M_* and *k*_cat_ values for AdoCbl of 194 μM and 6.5 min^−1^, respectively. Similarly, His_6_-MBP-CbiR hydrolyzes MeCbl, the active cofactor form used by MetH and other methyltransferases, to MeCbi-P (Fig. S4C), with comparable kinetic parameters (Fig. S4D), indicating that AdoCbl and MeCbl are equally suitable substrates for His_6_-MBP-CbiR.

### *A. muciniphila* CbiR can hydrolyze several different cobamides *in vitro*.

**FIG 4:**
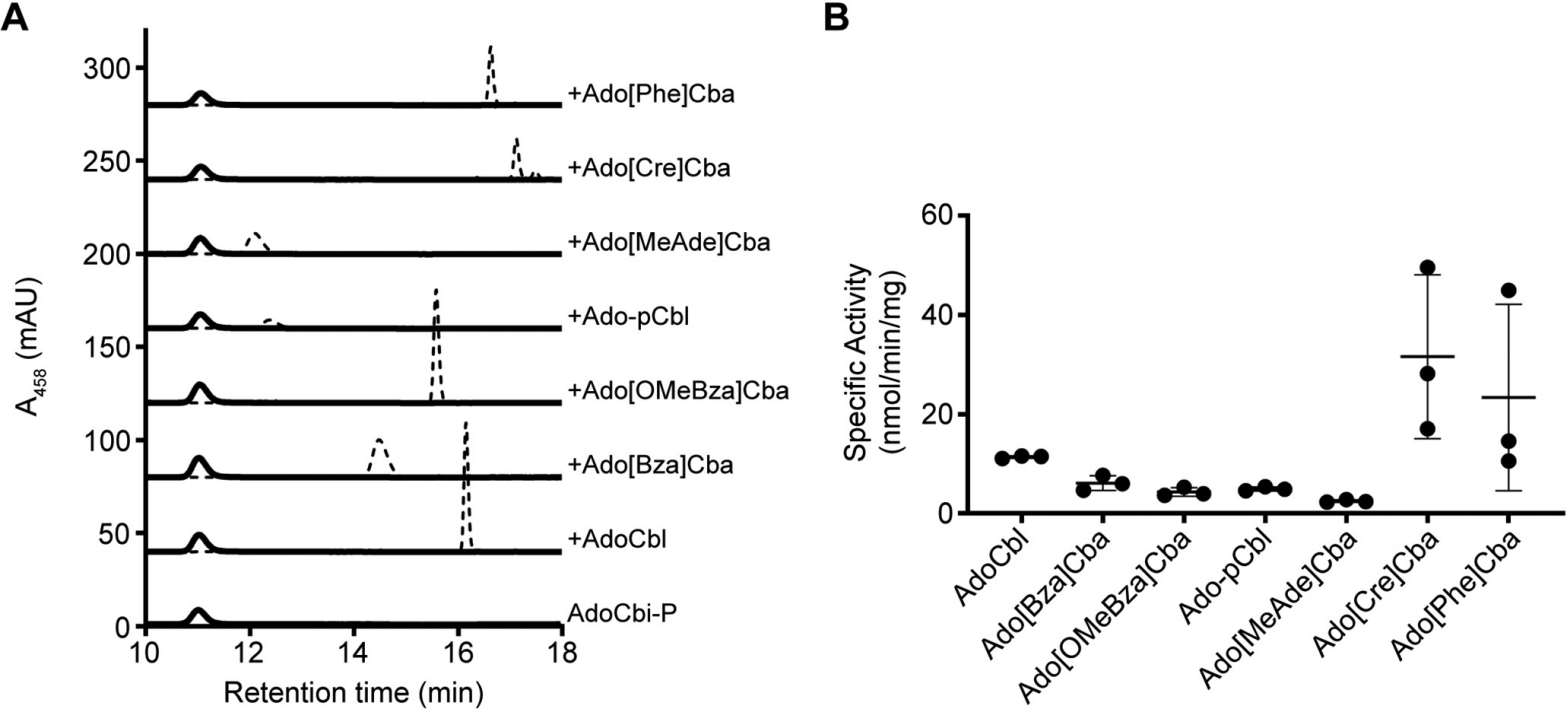
CbiR hydrolyzes many cobamides to form AdoCbi-P. (A) Results of HPLC analysis of *in vitro* reactions performed with different cobamides (10 μM), quenched after 18 h, are shown for reaction mixtures containing 0.1 μM His_6_-MBP-CbiR (solid lines) or without enzyme (dashed lines). A sample of purified AdoCbi-P is represented at the bottom. (B) Specific activity of His_6_-MBP-CbiR with different cobamide substrates. His_6_-MBP-CbiR (0.3 μM) was incubated with a 30 μM concentration of each cobamide individually, and the rate of AdoCbi-P production was determined based on HPLC measurements at three time points. The lines represent means and standard deviations of results from three independent experiments.

To determine the substrate selectivity of CbiR, His_6_-MBP-CbiR activity was measured *in vitro* with seven different cobamides. Adenosylated cobamides with purinyl or phenolyl lower ligands do not show UV-Vis spectra distinct from that of AdoCbi-P under the reaction conditions, and thus activity was measured by HPLC. Each cobamide was completely converted to AdoCbi-P following 18 h of incubation, demonstrating that all of the cobamides were substrates for CbiR (Fig. 4A). The specific activities of His_6_-MBP-CbiR with benzimidazolyl and purinyl cobamides were similar, with up to 4-fold differences among them, while higher activities were observed with phenolyl cobamides, though with greater variability between experiments (Fig. 4B). These *in vitro* results seen with CbiR appear to correlate with the remodeling activity found *in vivo* in *A. muciniphila* (Fig. 2C). The specific activities of His_6_-MBP-CbiR are similar to though slightly lower than that previously reported for CbiZ with Ado-pCbl (70 nmol/mg/min [59]), albeit under somewhat different reaction conditions.

### Expression of *cbiR* in E. coli enables expanded cobamide use.

**FIG 5:**
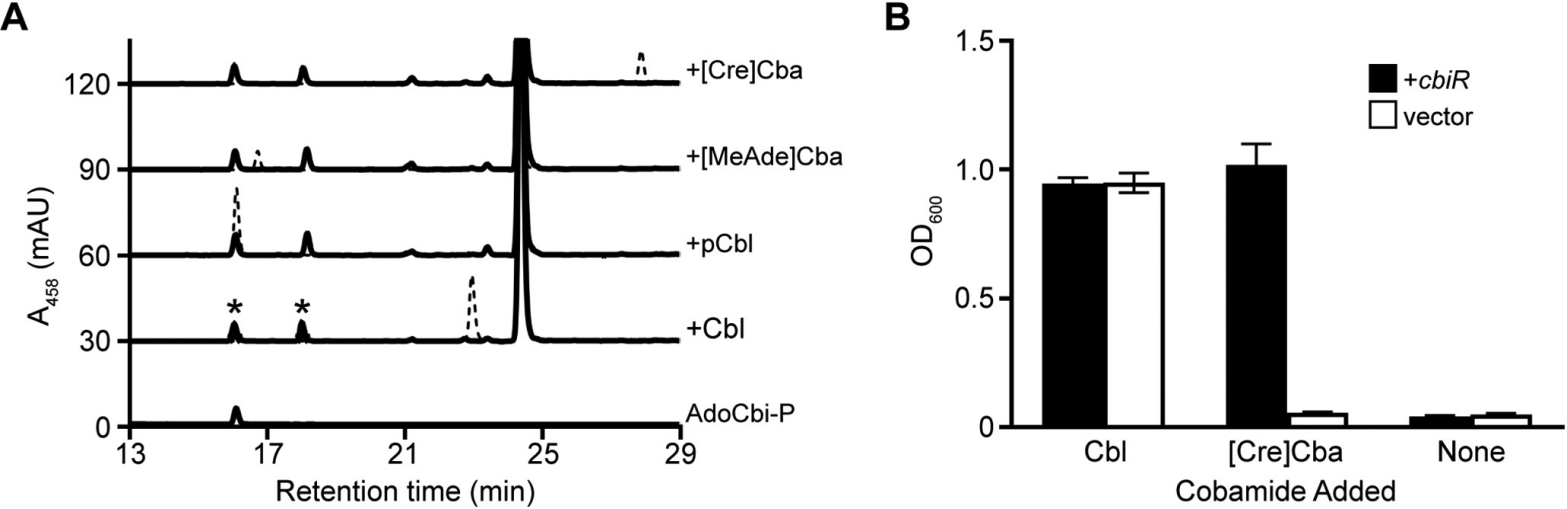
CbiR mediates cobamide remodeling in E. coli. (A) CbiR hydrolyzes cobamides in E. coli. *A. muciniphila cbiR* was expressed in an E. coli strain with the *cobTSU* operon and *cobC* gene deleted to prevent modification of cobamide hydrolysis products. Corrinoid extractions of E. coli strains carrying pETmini-*cbiR* (solid lines) or the pETmini empty vector (dashed lines) grown with 10 nM Cbl, pCbl, [MeAde]Cba, or [Cre]Cba were analyzed by HPLC. A sample of purified AdoCbi-P is represented at the bottom. Corrinoids labeled with asterisks were purified for MS analysis (Fig. S5). The large peak at 24.5 min corresponds to a flavin that is present in all of the corrinoid extractions. (B) Expression of *A. muciniphila cbiR* enables growth of E. coli on ethanolamine. Wild-type E. coli MG1655 harboring pETmini-*cbiR* (black bars) or the pETmini empty vector (white bars) was cultured in minimal medium containing ethanolamine as the sole nitrogen source and 1 μM DMB. OD_600_ measurements taken after 72 h of growth are shown for unsupplemented cultures and cultures supplemented with 100 nM Cbl or [Cre]Cba. Bars and error bars represent means and standard deviations, respectively, of results from three biological replicates.

Given that the product AdoCbi-P can be used as a precursor for construction of a different cobamide, we hypothesize that CbiR activity enables bacteria to remodel cobamides and therefore to gain access to cobamides in the environment that they otherwise might not be able to use. Because methods for targeted inactivation of genes in *A. muciniphila* have not been established, we used engineered E. coli strains to test this hypothesis. Like *A. muciniphila* strain Muc^T^, E. coli MG1655 cannot synthesize cobamides *de novo*, but its genome has the cobamide biosynthesis genes *cobT*, *cobS*, *cobU*, and *cobC*, which should allow E. coli to convert AdoCbi-P into a cobamide (81). We first tested whether *A. muciniphila* CbiR is functional in E. coli grown under aerobic conditions. Expression of untagged CbiR from a plasmid in a Δ*cobTSU* Δ*cobC* background did result in the loss of added Cbl, pCbl, [MeAde]Cba, and [Cre]Cba and the formation of two new corrinoid compounds (Fig. 5A). One of the products coeluted with AdoCbi-P (Fig. 5A), and MS analysis confirmed that the dominant ion matches the *m*/*z* values expected for Cbi-P (Fig. S5A). The second product gave *m*/*z* values consistent with Cbi (Fig. S5B), which likely formed intracellularly by hydrolysis of the phosphate group of AdoCbi-P. Neither product was detected in an E. coli strain containing the empty vector (Fig. 5A, dashed lines). Therefore, the activity of CbiR that we observed *in vitro* can be recapitulated in E. coli, even when it is cultured aerobically.

10.1128/mBio.02507-20.5 FIG S5 MS analysis of CbiR hydrolysis products extracted from E. coli. Results of MS analysis of the corrinoid products labeled with asterisks in Fig. 5A are shown for the peaks at (A) 16 min and (B) 18 min. The corrinoids were purified by HPLC and exposed to light to remove the adenosyl upper ligand prior to analysis by MS. The structures and *m*/*z* values predicted for Cbi-P and Cbi are shown for comparison. Download FIG S5, PDF file, 0.3 MB. Copyright © 2020 Mok et al. 2020 Mok et al. This content is distributed under the terms of the Creative Commons Attribution 4.0 International license.

Catabolism of ethanolamine in E. coli requires the cobamide-dependent enzyme ethanolamine ammonia lyase. Because this enzyme uses cobamides in the base-on conformation, in which a nitrogen atom in the lower ligand is coordinated to the cobalt atom (Fig. 1), it can use Cbl as a cofactor but is not functional with [Cre]Cba (82). We took advantage of this selectivity to design a cobamide remodeling-dependent growth assay in E. coli. In minimal medium supplemented with [Cre]Cba and 5,6-dimethylbenzimidazole (DMB; the lower ligand of Cbl), with ethanolamine as the sole nitrogen source, E. coli should be able to grow only if it can remodel [Cre]Cba to Cbl. Cbl, as expected, promoted growth of E. coli under this condition regardless of whether *cbiR* was present (Fig. 5B). In contrast, when [Cre]Cba was added, growth was observed only in the strain expressing *cbiR*, suggesting that CbiR activity enabled E. coli to convert [Cre]Cba into Cbl (Fig. 5B). A cobamide with a retention time and *m*/*z* values matching those of Cbl was detected in a corrinoid extraction of E. coli expressing *cbiR* grown with [Cre]Cba and DMB, confirming that cobamide remodeling to Cbl had occurred (Fig. S6). These results demonstrate that expression of CbiR expands the range of cobamides accessible to E. coli and suggest that cobamide remodeling may serve a similar purpose in *A. muciniphila*.

10.1128/mBio.02507-20.6 FIG S6 Cobamide remodeling of [Cre]Cba to Cbl in E. coli expressing *cbiR*. (A) Remodeling of [Cre]Cba to Cbl in E. coli. Wild-type strain MG1655 containing pETmini-*cbiR* was grown in 1 liter of minimal medium with ethanolamine supplemented with 100 nM [Cre]Cba and 1 μM DMB. Corrinoids were extracted and analyzed by HPLC. AdoCbl and Ado[Cre]Cba standards and purified AdoCbi-P are shown at the bottom. Comparison of the UV-Vis spectra of AdoCbl (dashed line) and the starred peak (solid line) are shown in the inset; spectra were normalized to each other at the local maxima at 458 nm to aid comparison. The spectrum of AdoCbl differs from that shown in Fig. 3E due to the acidic HPLC conditions. (B) MS analysis of the peak labeled with an asterisk in panel A. The corrinoid was purified by HPLC and exposed to light to remove the adenosyl upper ligand prior to analysis by MS. The structure and *m*/*z* values predicted for Cbl are shown for comparison. Download FIG S6, PDF file, 0.3 MB. Copyright © 2020 Mok et al. 2020 Mok et al. This content is distributed under the terms of the Creative Commons Attribution 4.0 International license.

### CbiR homologs are present in diverse bacteria.

**FIG 6:**
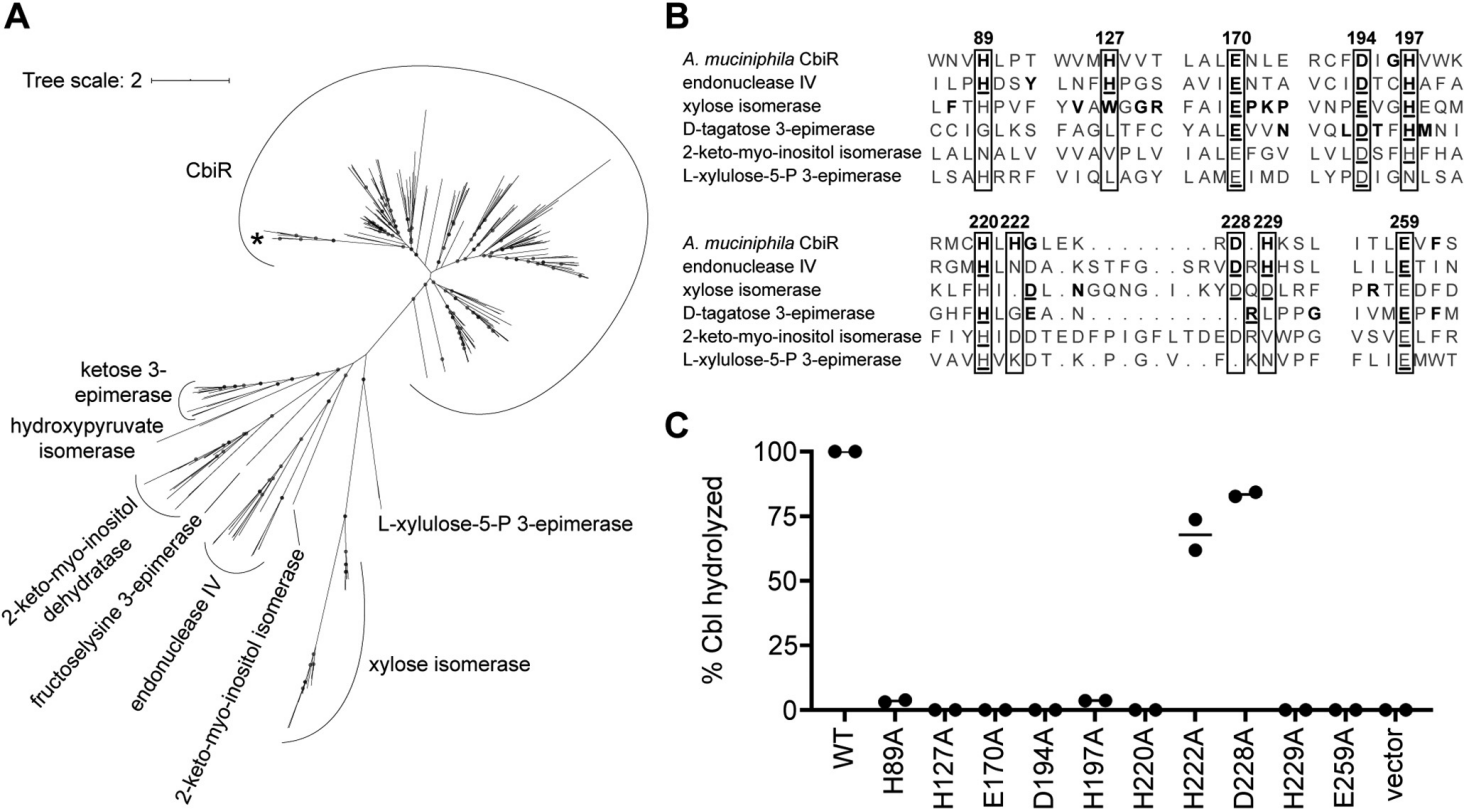
CbiR is a newly described member of the AP endonuclease 2 superfamily. (A) Maximum likelihood tree of CbiR homologs and members of the AP endonuclease 2 superfamily that have been experimentally characterized. *A. muciniphila* CbiR is indicated by an asterisk. CbiR homologs included in the tree were identified in a BLAST search queried with *A. muciniphila* CbiR with E values lower than 10^−14^ and encoded adjacent to and in the same orientation as one or more cobamide biosynthesis genes (Table S1). Superfamily member sequences are listed in Table S2. Gray circles overlaid on tree nodes represent bootstrap values of >95%. The scale bar corresponds to the average number of substitutions per site across the alignment. (B) Sequence alignment of regions containing highly conserved His, Asp, and Glu residues in *A. muciniphila* CbiR and representative sequences of biochemically and structurally characterized enzyme classes in the AP endonuclease 2 superfamily (E. coli endonuclease IV, Streptomyces rubiginosus xylose isomerase, Pseudomonas cichorii d-tagatose 3-epimerase, Bacillus subtilis 2-keto-myo-inositol isomerase, and E. coli l-xylulose-5-P 3-epimerase). Numbers correspond to positions in *A. muciniphila* CbiR. For CbiR, endonuclease IV, xylose isomerase, and d-tagatose (ketose) 3-epimerase, bolded residues represent conserved amino acids in the enzyme classes. Underlined residues indicate amino acids in the X-ray crystal structures that interact with the metal cofactor(s) and with the substrate in the case of *P. cichorii* d-tagatose 3-epimerase. (C) Mutational analysis of *A. muciniphila* CbiR. Corrinoids were extracted from cultures of E. coli Δ*cobTSU* Δ*cobC* strains carrying pETmini-*cbiR* (wild type [WT]), pETmini-*cbiR* with the specified alanine mutations, or the pETmini empty vector grown for 20 h with 75 nM Cbl and analyzed by HPLC. Shown on the *y*-axis is the combined amount of AdoCbi-P and AdoCbi as a percentage of the total adenosylated corrinoids extracted. Minimal amounts of CNCbl were detected in the mutant extractions, and the corresponding data were excluded from the analysis. The total amounts of intracellular corrinoid were similar between samples except for the WT and D228A samples, which had 4-fold-higher and 2.5-fold-higher levels, respectively. Lines show means of results from the two independent experiments.

Analysis of the sequence of CbiR revealed that it is not similar to that of CbiZ or V. cholerae CobS, the other enzymes known to have cobamide remodeling activity. Instead, as a member of the AP endonuclease 2 superfamily, CbiR is homologous to the enzymes endonuclease IV, 2-keto-myo-inositol dehydratase, and xylose isomerase and to other sugar isomerases and epimerases. A phylogenetic tree of this superfamily shows that the CbiR homologs identified by a BLAST search that are encoded in genomic loci containing cobamide biosynthesis genes form a single, distinct clade (Fig. 6A). Some of the biochemically characterized enzymes in the superfamily require metal cofactors for activity (63–80), and between one and three metal ions, such as Zn^2+^, Fe^2+^, Mg^2+^, and Mn^2+^, are found in nearly all X-ray crystal structures of enzymes from the superfamily (83–97). A metal cofactor may also be required for CbiR function; in addition to the inhibition by the metal chelator EDTA (Fig. S4B), CbiR homologs contain conserved His, Asp, and Glu residues that, in the characterized members of the superfamily, are involved in metal coordination (Fig. 6B) (86, 90, 93, 94, 96). Significant losses in activity have been observed in site-directed mutants of metal binding residues in AP endonuclease 2 superfamily members (87, 98–106). Similarly, E. coli strains expressing *cbiR* alleles with single mutations in many of these conserved residues had little to no AdoCbl hydrolysis activity (Fig. 6C), though effects of these mutations on enzyme folding or stability cannot be ruled out. These results demonstrate that CbiR shares both sequence and functional features common to the members of the AP endonuclease 2 superfamily and represents a new function within the superfamily.

10.1128/mBio.02507-20.7 TABLE S1 CbiR Blast homologs. Download Table S1, XLSX file, 0.03 MB. Copyright © 2020 Mok et al. 2020 Mok et al. This content is distributed under the terms of the Creative Commons Attribution 4.0 International license.

10.1128/mBio.02507-20.8 TABLE S2 Experimentally characterized members of AP endonuclease 2 superfamily. Download Table S2, XLSX file, 0.01 MB. Copyright © 2020 Mok et al. 2020 Mok et al. This content is distributed under the terms of the Creative Commons Attribution 4.0 International license.

Finally, we investigated the prevalence of CbiR across sequenced organisms by examining genomes with *cbiR* homologs. The *cbiR* gene commonly occurs in the *Akkermansia* genus, as a search of the 191 available genomes in the NCBI database found that 184 have a *cbiR* homolog. Additionally, 282 homologs of *A. muciniphila* CbiR with Expect (E) values below 10^−3^ were identified by BLAST in the genomes of 275 bacterial taxa and 1 archaeal taxon from diverse habitats, including aquatic environments, sewage, digesters, oil spills, bioreactors, soil, and human and animal hosts (see Table S1 in the supplemental material). While 76% are found in the phyla *Chlorobi*, *Chloroflexi*, and *Proteobacteria*, CbiR homologs are also present in 19 additional phyla (Fig. S3; see also Table S1), with relatively few in the PVC superphylum to which *A. muciniphila* belongs. Interestingly, nearly 20% of these taxa contain one or more homologs of *cbiZ* (Table S1), though it remains unknown whether the CbiR and CbiZ homologs in these strains function in cobamide remodeling. Similarly to *A. muciniphila*, 80% of all *cbiR* homologs are located adjacent to genes involved in cobamide biosynthesis (Table S1), suggesting that the majority of *cbiR* homologs function in cobamide remodeling.

## DISCUSSION

Cobamides are considered to be important modulators of mammalian gut ecosystems because they are involved in several metabolic pathways, their production is limited to a subset of prokaryotes, and their diverse structures are differentially accessible to different microbes (54, 107, 108). *A. muciniphila* has been shown to have positive effects on host metabolism, gut barrier function, and the inflammatory response (15–24), and yet knowledge of its metabolic and ecological roles in the gut remains incomplete. Previous studies showed that *A. muciniphila* strain Muc^T^ is unable to produce cobamides *de novo* (31) but can use pCbl produced by *E. hallii* or externally supplied Cbl for propionate production (26, 31). Here, while investigating the cobamide metabolism of *A. muciniphila* strain Muc^T^, we uncovered a novel cobamide remodeling activity and identified and characterized an enzyme capable of initiating this process, CbiR. This discovery adds new complexity to the understanding of the roles of *A. muciniphila* in the gut. Not only does *A. muciniphila* degrade mucin to provide nutrients to the gut microbiota (26, 27), but it is also capable of altering cobamide structure, potentially changing the cobamide composition of its environment.

As a member of the AP endonuclease 2 superfamily, CbiR likely contains a (β/α)_8_ TIM barrel domain (83–97), unlike the structures predicted for the CbiZ and CobS protein families (109). Thus, not only does CbiR catalyze a unique reaction, but it is also distinct from the other cobamide remodeling enzymes in sequence and likely in structure. Intriguingly, while CbiR differs in sequence from B_12_-binding domains in cobamide-dependent enzymes, the substrate-binding domains of many cobamide-dependent enzymes are comprised of a (β/α)_8_ TIM barrel structure, with the C-terminal face interacting with the cobamide cofactor (110–120). Given that CbiR is predicted to have a similar fold, it is possible that the cobamide binding mechanisms of CbiR and these cobamide-dependent enzymes share common features. The yet-to-be-discovered enzyme responsible for remodeling in algae may also be unique, as neither a P. lutheri transcriptome (121) nor the C. reinhardtii genome contains homologs of CbiR or CbiZ. It therefore appears that cobamide remodeling mechanisms have independently evolved multiple times. Together with the multiple pathways that exist for cobamide biosynthesis, transport, and precursor salvaging (32, 34, 35, 122–128), the addition of CbiR to the growing list of enzymes involved in cobamide metabolism highlights the importance of cobamide physiology in the evolution of bacteria.

In contrast to previously characterized cobamide remodeling enzymes and pathways, CbiR has promiscuous activity, hydrolyzing cobamides irrespective of their lower ligand structure. This activity differs from that of R. sphaeroides CbiZ, which does not hydrolyze Cbl *in vitro* (59), and V. cholerae CobS, which remodels neither Cbl nor [Cre]Cba (48). In those cases, the cobamide remodeling pathway does not act on a cobamide(s) that can function in its organism's metabolism. In contrast, *A. muciniphila* CbiR readily hydrolyzes pCbl, which functions as a cofactor for methionine synthesis and propionate metabolism in *A. muciniphila* and is the product of Cbi salvaging and cobamide remodeling in the bacterium itself. Thus, it is unclear how *A. muciniphila* prevents CbiR from continuing to hydrolyze pCbl after it is formed via cobamide remodeling. It is possible that pCbl is sequestered intracellularly by binding to MetH or other cobamide-dependent enzymes, such as may have occurred in our ethanolamine-based growth assay in E. coli in which *cbiR* was constitutively expressed. Alternatively, CbiR activity could be coupled to cobamide uptake, as has been suggested previously for CbiZ (59, 129). Indeed, similarly to some *cbiZ* homologs, 25% of *cbiR* homologs are located adjacent to genes encoding putative transport proteins, including in *A. muciniphila* strain Muc^T^ (see Fig. S3 in the supplemental material). Remodeling in *D. mccartyi* strain 195 shows substrate promiscuity similar to that seen with *A. muciniphila* with respect to the ability to act on numerous, structurally diverse cobamides (56), but the molecular basis of this promiscuity is unclear because its genome carries seven *cbiZ* homologs, none of which has been biochemically characterized. Aside from its activity on pCbl, the broad substrate range of CbiR may benefit *A. muciniphila* by enabling the bacterium to utilize a greater number of the cobamides present in its environment.

The discovery that *A. muciniphila* remodels cobamides leads us to reexamine its ecological roles in the gut. CbiR is found in all 75 of the recently sequenced *A. muciniphila* strains from the human and mouse gut, including the 26 strains that contain the *de novo* cobamide biosynthesis pathway (31, 130). Thus, like cobamide-dependent metabolism (31), cobamide remodeling appears to be nearly universal in *A. muciniphila*. Further, the role of *A. muciniphila* in the gut may be flexible, ranging from producing cobamides *de novo* to remodeling cobamides produced by other microbes, depending on which strains inhabit an individual. Notably, the end product of cobamide remodeling in *A. muciniphila*, pCbl, was the third most abundant corrinoid detected in the human gut in a study of human subjects residing at a single geographic location (42). Interestingly, that study also presented evidence that cobamide remodeling occurs in the human gut, as individuals supplemented with high levels of Cbl showed transiently increased levels of Cbi and of the specific purinyl and phenolyl cobamides that were present in the gut prior to Cbl supplementation. It is possible that *A. muciniphila* is involved in this remodeling activity and contributes to the pool of pCbl in the gut. This, in turn, could modulate the growth or metabolism of other cobamide-requiring bacteria that rely on particular cobamides for their metabolic needs. CbiR may therefore not only expand access to the cobamides available to *A. muciniphila* but also affect those accessible to other bacteria in the gut. Further, homologs of CbiR are found in at least 276 other microbial taxa and may function similarly in those microbes that inhabit diverse environments. The addition of CbiR to the cobamide remodeling enzymes that have been characterized to date—CbiZ, certain CobS homologs, and the enzyme(s) responsible for remodeling in algae—suggests that cobamide remodeling is more widespread than previously thought.

## MATERIALS AND METHODS

### Media and growth conditions.

Akkermansia muciniphila strain Muc^T^ (DSM 22959, ATCC BAA-835) was cultivated at 37°C in a vinyl anaerobic chamber (Coy Laboratory Products Inc.) under an atmosphere of approximately 10% CO_2_, 3% H_2_, and 87% N_2_. A synthetic version of a basal mucin-based medium, in which mucin was replaced by soy peptone (16 g/liter), l-threonine (4 g/liter), glucose (2 g/liter), and *N*-acetylglucosamine (2 g/liter), was supplemented with 1% noble agar and used as a solid medium (21). This synthetic medium also contained l-methionine (125 mg/liter) and omitted rumen fluid. M8 defined medium, developed by Tramontano et al. (131), was used for liquid culturing. We found that the concentration of mucin in this medium (0.5%) was able to abrogate the requirement of methionine addition to the medium for *A. muciniphila* growth. Lowering the concentration to 0.25% resulted in methionine-deplete conditions for *A. muciniphila*, and supplementation with methionine or cobamides restored robust growth. This mucin concentration was used for the MetH-dependent growth assays. However, batch-to-batch variations were seen with mucin such that formulations of media that supported robust growth while remaining methionine-deplete could not always be achieved. Cobamides were omitted from all growth media except when specified. For MetH-dependent growth assays, methionine was omitted from M8 medium.

Escherichia coli was cultured at 37°C with aeration in LB medium for cloning, protein expression, and assessing CbiR hydrolytic activity. Ethanolamine-based growth experiments used medium from Scarlett and Turner (38), with B_12_ omitted. Media were supplemented with antibiotics at the following concentrations when necessary: kanamycin, 25 mg/liter (pETmini); ampicillin, 100 mg/liter (pET-His_6_-MBP); chloramphenicol, 20 mg/liter (pLysS).

For all *in vivo* experiments involving corrinoids, culture media were supplemented with cyanylated cobamides or (CN)_2_Cbi, which are adenosylated following uptake into cells.

### Genetic and molecular cloning techniques.

The entire *A. muciniphila cbiR* open reading frame, except the start codon, was cloned into a modified pET16b vector (132) with N-terminal His_6_ and MBP tags added for protein purification. For analysis of CbiR activity in E. coli, a minimized 3-kb derivative of pET28a (pETmini) containing a kanamycin resistance (Kan^r^) marker, pBR322 origin, and *rop* gene was used. A constitutive promoter (BBa_J23100; iGEM) and ribosome binding site (RBS) (BBa_B0034) were inserted into the vector for expression in E. coli MG1655-based strains (complete sequence, TTGACGGCTAGCTCAGTCCTAGGTACAGTGCTAGCGAATTCATACGACTCACTATAAAAGAGGAGAAA) and *A. muciniphila cbiR* was cloned downstream. Site-directed mutations were introduced into *cbiR* by PCR. All cloning was done by Gibson assembly with E. coli XL1-Blue cells (133).

Construction of the E. coli MG1655 Δ*cobTSU* Δ*cobC* strain was accomplished using λ Red-based recombination (134) and phage P1 transduction (135). An MG1655 Δ*cobTSU*::Kan^r^ operon deletion was constructed by λ Red-based recombination. The Δ*cobC*::Kan^r^ allele was transduced into MG1655 via P1 transduction from E. coli strain JW0633-1, which was obtained from the Keio collection (136). Kan^r^ cassettes were removed by recombination of the flanking FLP recombination target (FRT) sites as described previously (134).

### Chemical reagents.

Porcine gastric mucin was purchased from MilliporeSigma (M1778). AdoCbl (coenzyme B_12_), MeCbl, CNCbl, and (CN)_2_Cbi were purchased from MilliporeSigma.

### Cobamide synthesis, adenosylation, and quantification.

All other cyanylated cobamides used in the study were purified from bacterial cultures, and cobamides were adenosylated and purified as previously described (50, 137). Cyanylated and adenosylated cobamides were quantified as previously described (50). MeCbl was quantified using an extinction coefficient of ε_519_ = 8.7 mM^−1 ^cm^−1^ (138). AdoCbi-P and MeCbi-P were quantified using the dicyanylated corrinoid extinction coefficient ε_580_ = 10.1 mM^−1 ^cm^−1^ following conversion to (CN)_2_Cbi-P by incubation with 10 mM KCN in the presence of light (139).

### *A. muciniphila* MetH-dependent growth assay.

*A. muciniphila* was precultured for 48 h in M8 medium supplemented with 125 mg/liter methionine. Cells were pelleted by centrifugation, washed twice with phosphate-buffered saline (PBS), and diluted into 80 μl M8 medium to an optical density at 600 nm (OD_600_) of 0.02 in a 384-well plate (Nunc) with various concentrations of cobamides and Cbi. The wells were sealed (ThermalSeal RTS; Excel Scientific), and the plate was incubated at 37°C in a BioTek Epoch 2 microplate reader. OD_600_ values were measured at regular intervals during growth.

### E. coli ethanolamine-dependent growth assay.

E. coli was precultured 16 h in ethanolamine medium (38) supplemented with 0.02% ammonium chloride. Cells were pelleted by centrifugation, washed three times with 0.85% NaCl, and diluted to an OD_600_ of 0.025 in 200 μl ethanolamine medium with the specified cobamide additions in a 96-well plate (Corning). The wells were sealed (Breathe-Easy; Diversified Biotech), and OD_600_ was monitored at 37°C in a BioTek Synergy 2 microplate reader with shaking.

### Protein expression and purification.

His_6_-MBP-CbiR was expressed in E. coli Rosetta(DE3) pLysS. Cells were grown to an OD_600_ of 0.4 at 37°C, and expression was induced with 1 mM IPTG (isopropyl-β-d-thiogalactopyranoside) for 6 h at 30°C. Cells were lysed by sonication in a buffer containing 20 mM sodium phosphate, 300 mM NaCl, 10 mM imidazole (pH 7.4), 0.5 mM phenylmethylsulfonyl fluoride (PMSF), 1 μg/ml leupeptin, 1 μg/ml pepstatin, and 1 mg/ml lysozyme. The protein was purified from the clarified lysate using HisPur nickel-nitrilotriacetic acid (Ni-NTA) resin (Thermo Scientific) and eluted with 250 mM imidazole. Purified protein was dialyzed into a buffer containing 25 mM Tris-HCl (pH 8.0), 300 mM NaCl, and 10% glycerol and stored at −80°C.

### His_6_-MBP-CbiR *in vitro* reactions.

Due to light sensitivity of the compounds, all work involving adenosylated cobamides or MeCbl was performed in the dark or under red or dim white light. Unless specified, the *in vitro* reactions were performed at 37°C in a vinyl anaerobic chamber with the atmosphere described above. The components of the reaction mixtures were 50 mM Tris buffer, 0.3 μM purified His_6_-MBP-CbiR, 1 mM DTT, and various concentrations of a cobamide. To prepare the Tris buffer, Tris base was dissolved and equilibrated within the anaerobic chamber. Prior to each experiment, the pH was adjusted with NaOH to account for acidification by the CO_2_ present in the atmosphere of the chamber. The pH was adjusted to 8.8 to 8.9 at room temperature (approximately 24°C), corresponding to a predicted pH range of 8.45 to 8.55 at 37°C. Protein concentrations were determined by absorbance at 280 nm (*A*_280_).

A BioTek Epoch 2 microplate reader and half-area UV-Star 96-well microplates (Greiner Bio-One) were used for assays monitoring the reaction by absorbance. For these assays, separate 2× solutions of AdoCbl and His_6_-MBP-CbiR were prepared in a buffer containing 50 mM Tris and 1 mM DTT. A frozen aliquot of His_6_-MBP-CbiR was thawed inside the anaerobic chamber prior to dilution. The 2× AdoCbl solution was preincubated at 37°C for 60 min, while the 2× CbiR solution was preincubated at 37°C for 20 min. Volumes of 60 μl each of 2× AdoCbl and 2× His_6_-MBP-CbiR were then mixed in a 96-well plate, with 100 μl transferred to a new well prior to performing measurements in the plate reader. Assays with MeCbl were prepared similarly.

Absorbances measured over time at 534 and 527 nm were used to monitor the conversion of AdoCbl to AdoCbi-P and of MeCbl to MeCbi-P, respectively. To enable conversion of *A*_534_ values into moles of AdoCbl and of *A*_527_ values into moles of MeCbl, extinction coefficient values corresponding to AdoCbl and MeCbl and to purified AdoCbi-P and MeCbi-P were determined in a buffer containing 50 mM Tris (pH 8.8) and 1 mM DTT as follows: ε_534_ (AdoCbl) = 7.8 mM^−1 ^cm^−1^, ε_527_ (MeCbl) = 8.0 mM^−1 ^cm^−1^, ε_534_ (AdoCbi-P) = 1.3 mM^−1 ^cm^−1^, ε_527_ (MeCbi-P) = 2.7 mM^−1 ^cm^−1^.

For reactions performed with adenosylated cobamides with different lower ligands monitored by HPLC, cobamides were mixed at 60 μM with 50 mM Tris buffer and 1 mM DTT and equilibrated to 37°C. His_6_-MBP-CbiR was equilibrated to 37°C at 0.6 μM in a buffer containing 50 mM Tris and 1 mM DTT. Each cobamide and His_6_-MBP-CbiR were mixed in equal volumes, and the reaction mixtures were incubated at 37°C. At three different time points, 100 μl of the reaction mixture was removed and mixed with 5 μl of 600 mM EDTA to quench the reaction. The protein was removed from samples using Nanosep 10K centrifugal devices (Pall) prior to injection onto the HPLC system. AdoCbi-P levels in the samples were quantified by HPLC by comparison to a standard curve generated with known quantities of purified AdoCbi-P. For reactions involving incubations of 4 to 18 h, initial equilibration at 37°C was not performed.

### Corrinoid extraction.

Cbi salvaging and cobamide remodeling were assessed in *A. muciniphila* by cultivation in M8 medium supplemented with 10 nM Cbi and cobamide, respectively, for 72 h. CbiR cobamide hydrolytic activity with different cobamides in E. coli was monitored using the MG1655 Δ*cobTSU* Δ*cobC* strain cultivated in LB medium supplemented with 10 nM cobamide for 16 h. Cobamide remodeling in E. coli MG1655 was assessed by cultivation in ethanolamine medium supplemented with 100 nM [Cre]Cba and 1 μM DMB for 94 h. CbiR mutants were analyzed in E. coli MG1655 Δ*cobTSU* Δ*cobC* by culturing in LB medium supplemented with 75 nM Cbl for 20 h.

Cyanation of corrinoids extracted from cells for the experiments whose results are shown in Fig. 2A and C (see also Fig. S1 and S2 in the supplemental material) was performed as previously described (56), with 5,000 corrinoid molar equivalents of KCN added. For extractions of adenosylated corrinoids (Fig. 5A and 6C; see also Fig. S5 and S6), cell lysis was performed similarly, with KCN omitted; following removal of cellular debris by centrifugation, deionized water was added to the supernatant to decrease the methanol concentration to 10%. Solid-phase extraction of cyanylated and adenosylated corrinoids with Sep-Pak C_18_ cartridges (Waters) was performed as described previously (37). Samples were dried, resuspended in 200 μl deionized water (pH 7), and filtered with 0.45-μm-pore-size filters (Millex-HV; Millipore) or Nanosep 10K centrifugal devices prior to analysis by HPLC. For extractions involving adenosylated cobamides, all steps were performed in the dark or under red or dim white light.

### HPLC and MS analysis.

Corrinoids were analyzed on an Agilent 1200 series HPLC system equipped with a diode array detector. For the experiments whose results are shown in Fig. 2A and C, 3B, and 4A and B, an Agilent Zorbax SB-Aq column (5-μm pore size, 4.6 by 150 mm) was used as previously described (method 2 [58]). For the experiments whose results are shown in Fig. 5A and 6C (see also Fig. S6A), an Agilent Zorbax Eclipse Plus C_18_ column (5-μm pore size, 9.4 by 250 mm) was used with the following method: solvent A, 0.1% formic acid–deionized water; solvent B, 0.1% formic acid–methanol; 2 ml/min at 30°C; 18% solvent B for 2.5 min followed by a linear gradient of 18% to 60% solvent B over 28.5 min.

An Agilent 1260 series fraction collector was used for HPLC purification of corrinoids and CbiR reaction products. Purification of CN-pCbl from *A. muciniphila* was performed using the Zorbax SB-Aq column with the method described above. Purification of AdoCbi-P and α-ribazole from the hydrolysis of AdoCbl by His_6_-MBP-CbiR was performed in two steps. AdoCbi-P and α-ribazole were first separated and collected on a Zorbax Eclipse XDB C_18_ column (5-μm pore size, 4.6 by 150 mm) using the following method: solvent A, 10 mM ammonium acetate (pH 6.5); solvent B, 100% methanol; 1 ml/min at 30°C; 0% solvent B for 2 min followed by a linear gradient of 0% to 15% solvent B over 1.5 min, 15% to 35% over 6.5 min, 35% to 70% over 2 min, and 70% to 100% over 2 min. Each compound was subsequently run and collected on the Zorbax SB-Aq column with the method described above. Purification of MeCbi-P from the *in vitro* hydrolysis of MeCbl was performed using the Zorbax SB-Aq column with the method described above. The purification of adenosylated hydrolysis products of CbiR from E. coli was performed with two rounds of collection; AdoCbi-P and AdoCbi were first separated and collected on the Zorbax Eclipse Plus C_18_ column with the method above, and then each compound was run and collected on the Zorbax SB-Aq column using the method described. AdoCbl remodeled from [Cre]Cba in E. coli was purified using the Zorbax Eclipse Plus C_18_ column with the method described above. Collected compounds were desalted with Sep-Pak C_18_ cartridges.

MS analysis was performed on a Bruker linear quadrupole ion trap coupled to a Fourier transform ion cyclotron (LTQ-FT) mass spectrometer at the QB3/Chemistry MS Facility (University of California, Berkeley [UC Berkeley]). Prior to MS analysis, the purified adenosylated and methylated corrinoids were exposed to light to remove the adenosyl and methyl upper ligands, respectively.

### Phylogenetic analysis.

A total of 282 homologs of *A. muciniphila* strain Muc^T^ CbiR were identified by BLAST, with E values lower than 10^−3^ (see Table S1 in the supplemental material; sequences from other strains of *A. muciniphila* are excluded). A subset of 203 sequences with E values lower than 10^−14^ and whose encoding genes were located adjacent to and in the same orientation as a cobamide biosynthesis gene(s) were chosen for phylogenetic analysis performed with the AP endonuclease 2 superfamily (pfam 01261) (Table S1). Sequences were clustered at 0.95 using CD-HIT to reduce the CbiR homolog sequence set by removing subspecies sequence diversity (140). This final set of 178 sequences and *A. muciniphila* CbiR were used to infer a phylogenetic tree with experimentally characterized members of the AP endonuclease 2 superfamily (Table S2). The sequences were aligned with MAFFT (141), and positions with 95% or greater gaps were removed by the use of trimAl (142). A maximum likelihood tree (presented in Fig. 6A) was inferred from this alignment using IQ-TREE v1.6.12 (143) with 1,500 ultrafast bootstraps and visualized in iTOL (144).
