# Supplementary figures and images for: Identification of a Novel Cobamide Remodeling Enzyme in the Beneficial Human Gut Bacterium Akkermansia muciniphila
Source: mBio. 2020 Dec 8;11(6):e02507-20. doi: 10.1128/mBio.02507-20 (PMC7733943; doi:10.1128/mBio.02507-20)

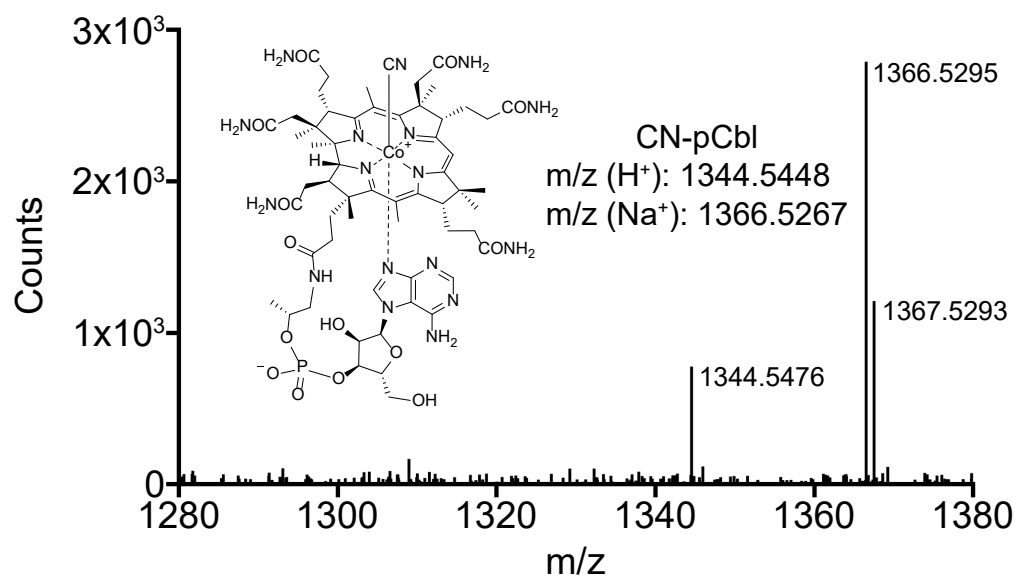

Supplement: FIG S1 [file SM-MBIO200008sf1.pdf]

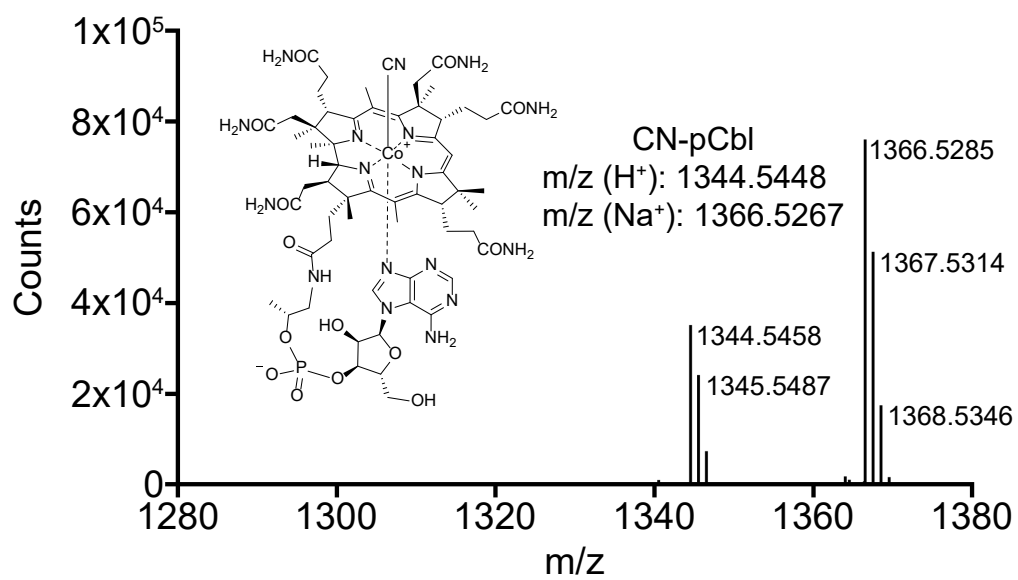

Supplement: FIG S2 [file SM-MBIO200008sf2.pdf]

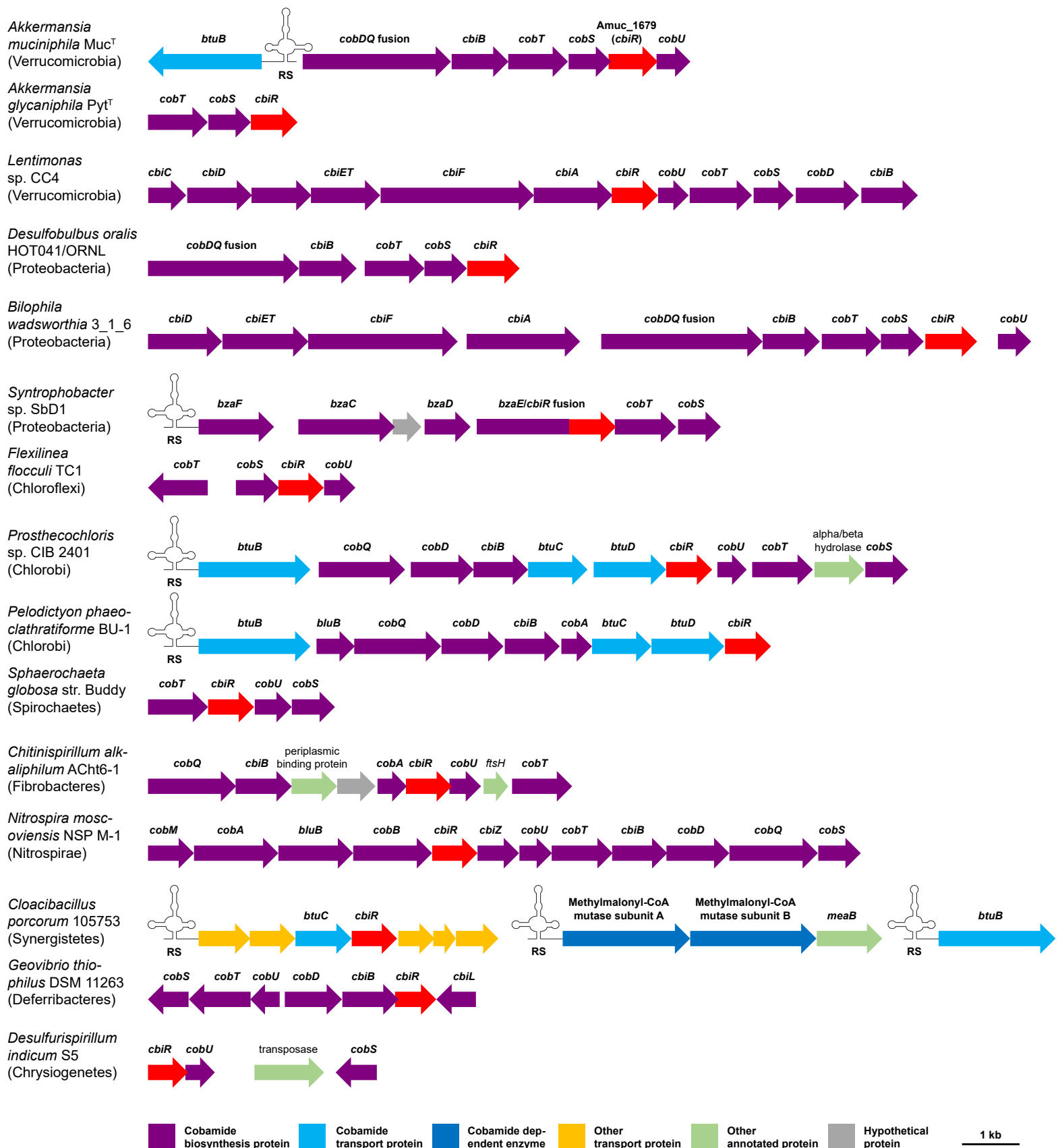

Supplement: FIG S3 [file SM-MBIO200008sf3.pdf]

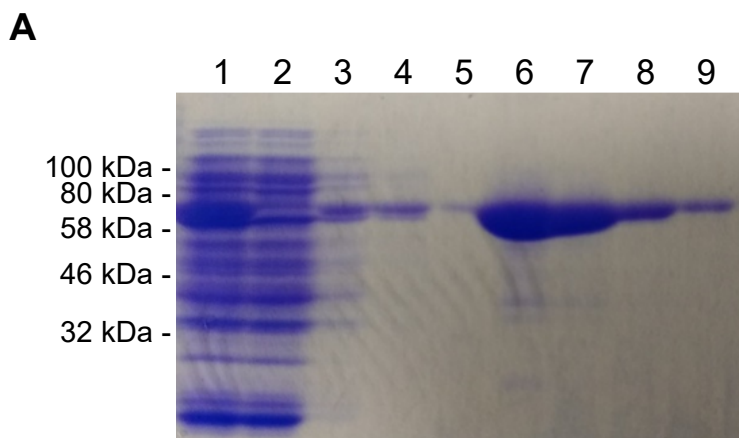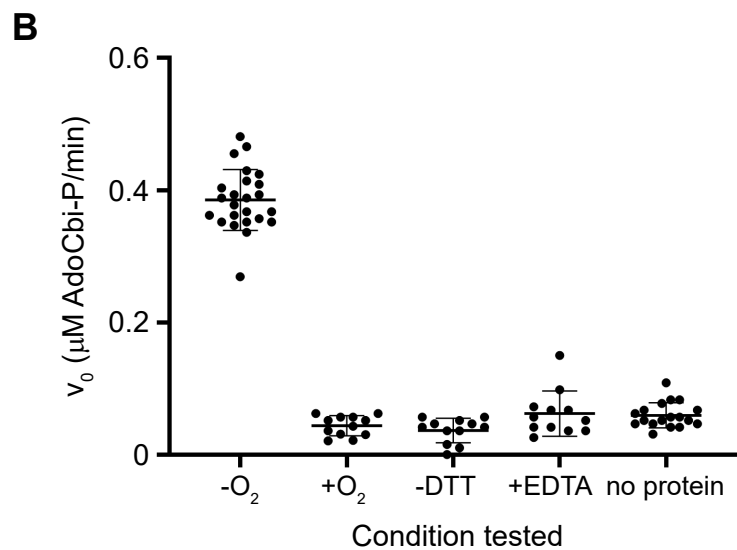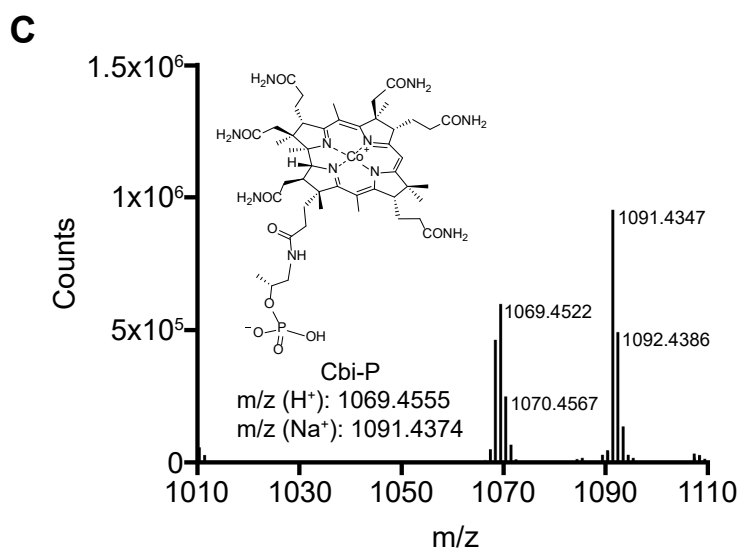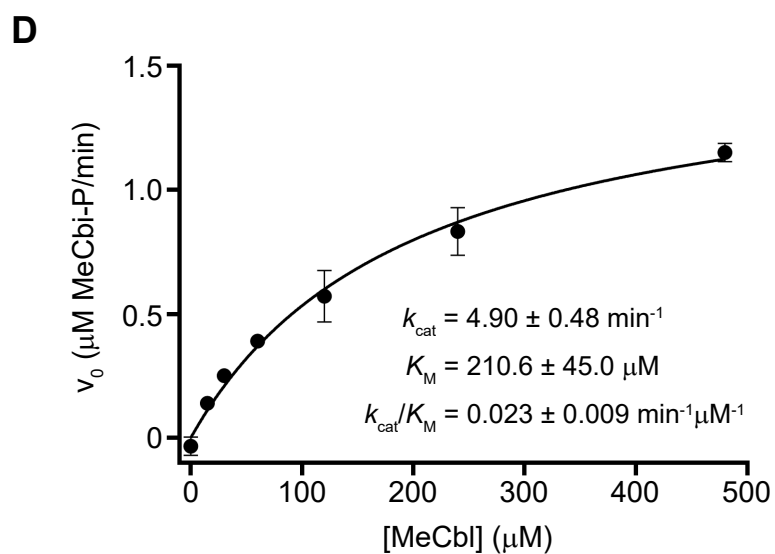

Supplement: FIG S4 [file SM-MBIO200008sf4.pdf]

**A**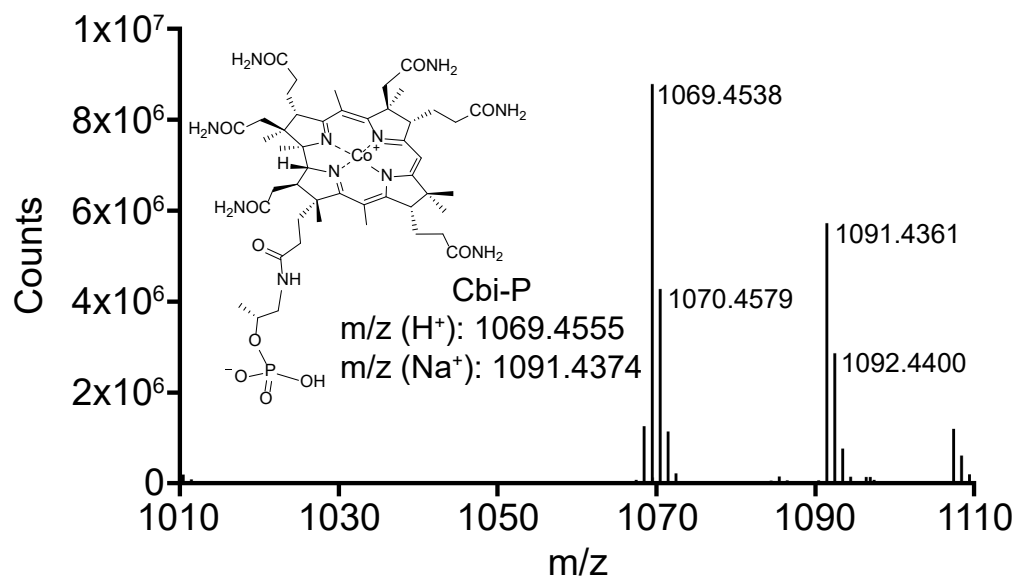**B**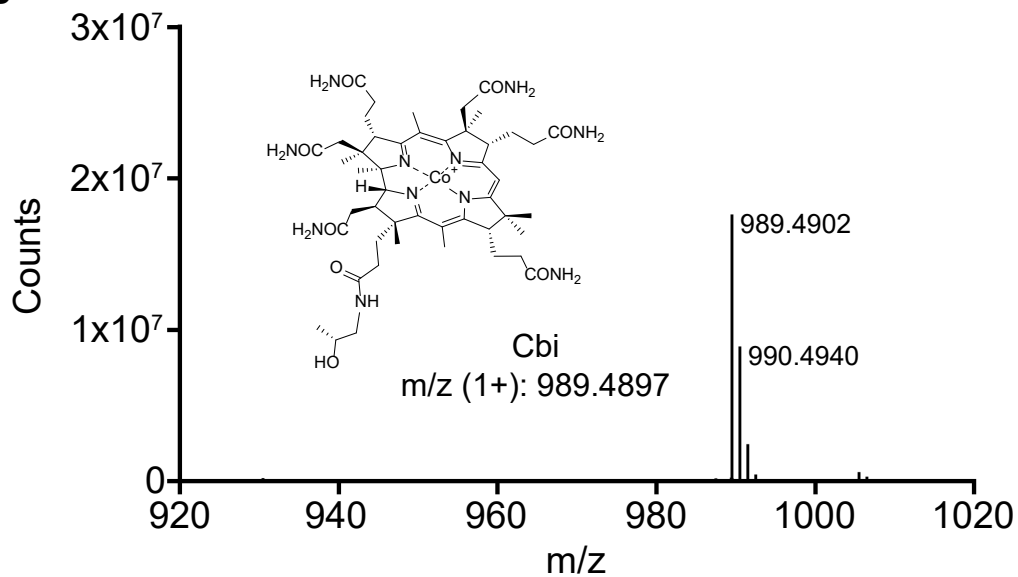

Supplement: FIG S5 [file SM-MBIO200008sf5.pdf]

**A**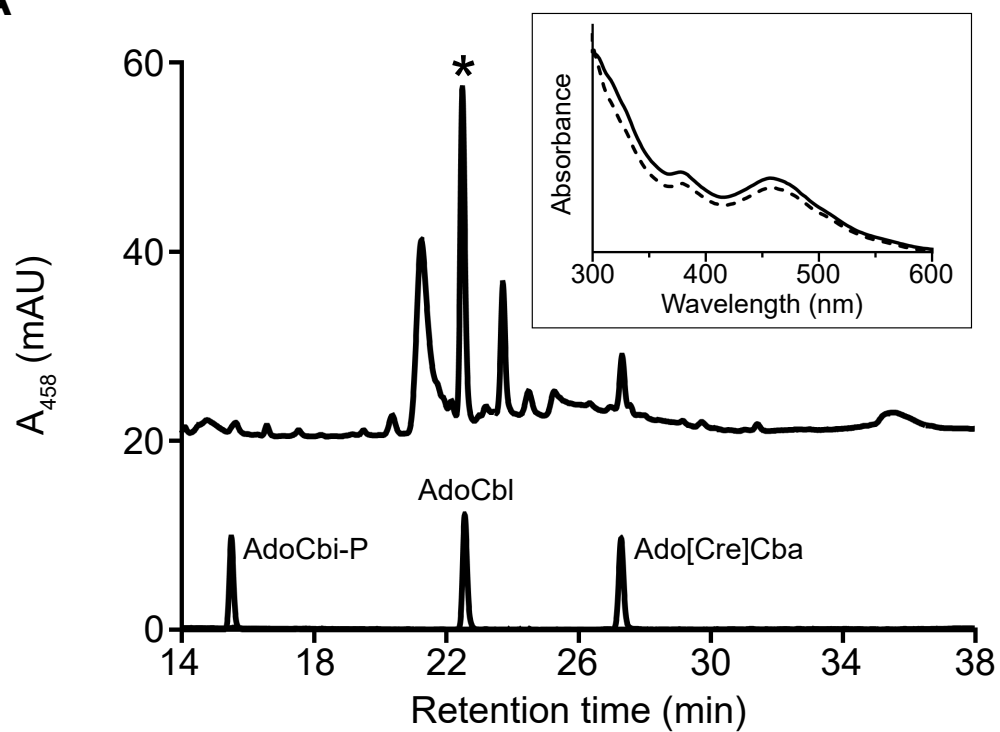**B**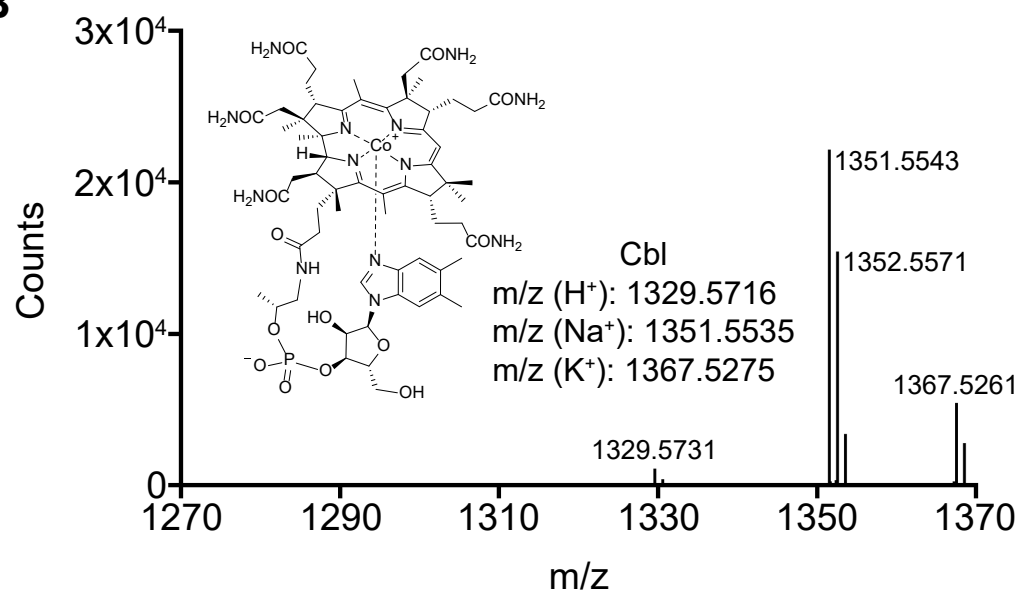

Supplement: FIG S6 [file SM-MBIO200008sf6.pdf]
